# Supplementary material for: ICOS costimulation is indispensable for the differentiation of T follicular regulatory cells
Source: Life Sci Alliance. 2023 Feb 8;6(4):e202201615. doi: 10.26508/lsa.202201615 (PMC9909462; doi:10.26508/lsa.202201615)
Supplement: Supplementary file 1 [file LSA-2022-01615_SdataF5.pdf]

Raw data for Fig. 5F

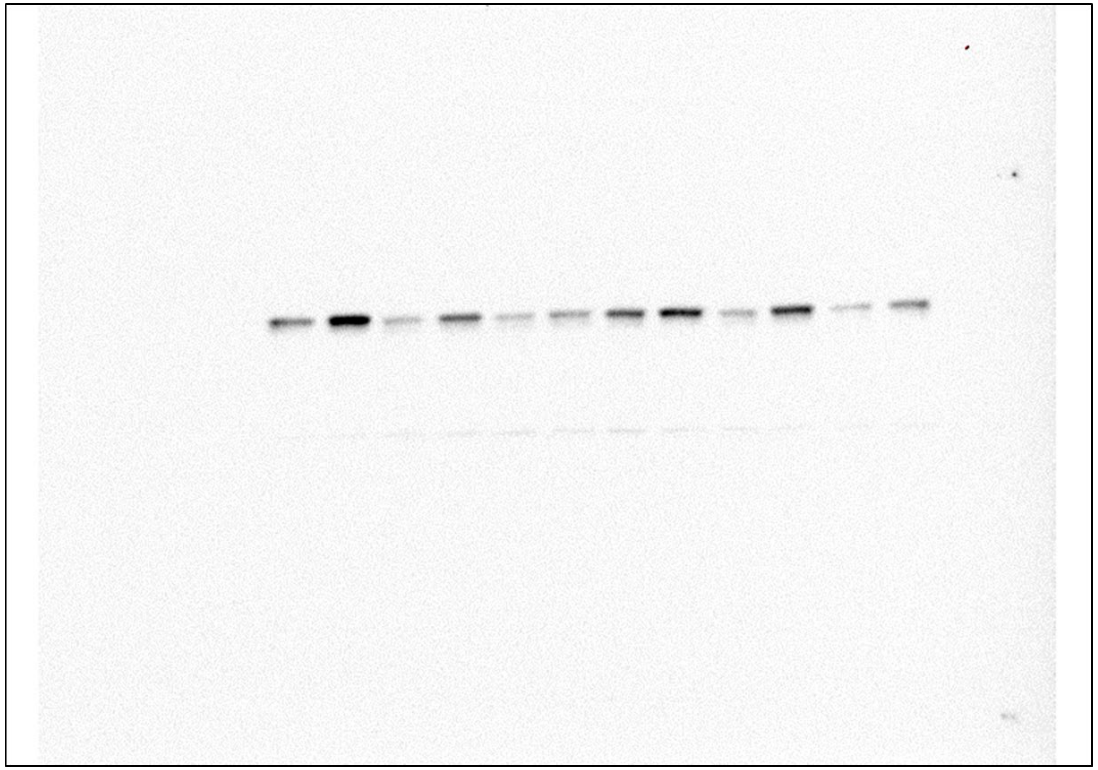

Cytoplasmic NFAT2
Acquired on Bio-Rad ChemiDoc imaging system
Exposure time : 27s

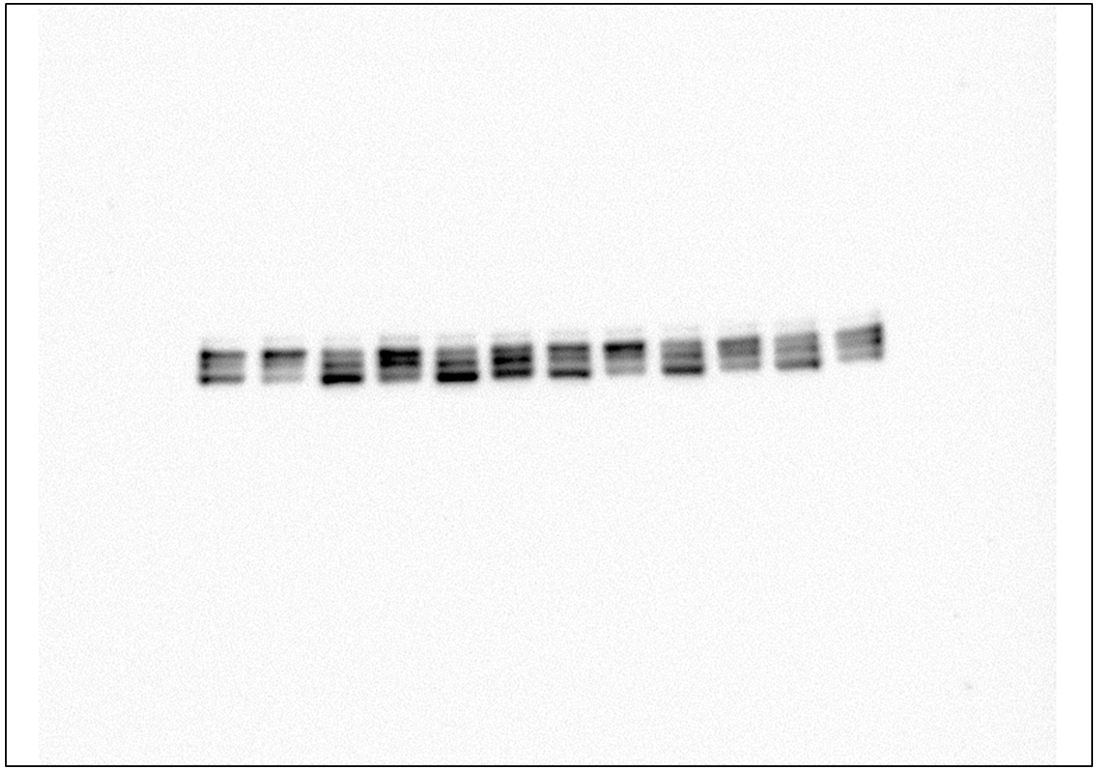

Nuclear NFAT2
Acquired on Bio-Rad ChemiDoc imaging system
Exposure time : 9s
