# Supplementary material for: ICOS costimulation is indispensable for the differentiation of T follicular regulatory cells
Source: Life Sci Alliance. 2023 Feb 8;6(4):e202201615. doi: 10.26508/lsa.202201615 (PMC9909462; doi:10.26508/lsa.202201615)
Supplement: Supplementary file 2 [file LSA-2022-01615_TableS1.pdf]

Reference	Genes
Mi, H., *et al*. (2021)	*Actc1, Acta1, Acta2, Actg2, Adssl1, Adss, Edn1, Myh1, Myh2, Myh3, Myh6, Myh7, Myh7b, Myh8, Myh13*
Mognol, G.P., *et al*. (2016)	*Bcl2, Fasl, Myc, Ccna2, Ccnd1, Ccnd3, Cdkn1a, Tnf, Tnfsf10, Ddias*
Herman-Kleiter, N. and Baier, G. (2010)	*Il2, Ifng, Il17a, Il17f, Il22, Il4, Il5, Il13, Il21*
Vaeth, M., *et al.* (2014)	*Cxcr5*

Supplementary table 1 List of NFAT target genes
